# The Effects of Pioglitazone on Biochemical Markers of Bone Turnover in the Patients with Type 2 Diabetes

**DOI:** 10.1155/2013/290734

**Published:** 2013-06-16

**Authors:** Wen-hua Xiao, Yan-rong Wang, Wen-fang Hou, Chao Xie, Hai-ning Wang, Tian-pei Hong, Hong-wei Gao

**Affiliations:** ^1^Department of Endocrinology, Peking University Third Hospital, Beijing 100191, China; ^2^Department of Endocrinology and Metabolism, Peking University Third Hospital, Beijing 100191, China

## Abstract

*Aim.* To investigate whether pioglitazone had detrimental effects on biochemical markers of bone turnover in patients with type 2 diabetes (T2DM). *Methods.* Seventy patients with T2DM were included in this study. The patients remained on their previous antihyperglycemic therapies during the trial. Pioglitazone was then added on their regimen for 3 months. *Results.* After 3 months of treatment with pioglitazone, the levels of fasting blood glucose and HbA_1c_ were significantly decreased (7.9 ± 1.5 mmol/L versus 9.1 ± 1.6 mmol/L and 7.1 ± 1.0% versus 8.2 ± 1.4%, resp., *P* < 0.01), compared with baseline in the overall patients. Serum concentrations of P1NP and BAP were significantly decreased from baseline (45.0 ± 20.0 **μ**g/L versus 40.6 ± 17.9 **μ**g/L and 13.23 ± 4.7 **μ**g/L versus 12.3 ± 5.0 **μ**g/L, resp., *P* < 0.01) in female group, but not in male group. The serum levels of OC and CTX were unchanged in both female and male subgroups. In addition, the levels of serum BAP and P1NP were significantly decreased after pioglitazone treatment in postmenopausal subgroup, comparing with baseline. *Conclusion.* Pioglitazone inhibits bone formation and does not seem to affect bone resorption. Postmenopausal female patients rather than premenopausal or male patients are particularly vulnerable to this side effect of pioglitazone.

## 1. Introduction 

Recent studies have shown that type 2 diabetes (T2DM) is associated with abnormality in bone metabolism including bone loss and osteoporosis [1–4]. The risk of fragility fracture, in particular of the hip, proximal humerus, and foot, was also increased in diabetic patients [3, 5]. Thiazolidinediones (TZDs), selective agonists of peroxisome proliferators-activated receptor (PPAR-*γ*), are worldwide prescribed oral antihyperglycemic agents. The safety of TZDs, however, is questioned because of their adverse effects on bone metabolism and high risk of fracture in patients receiving the treatment of TZDs. The data from ADOPT (A Diabetes Outcome Progression Trial) suggested that the incidence of fractures in lower and upper limbs significantly increased in diabetic women treated with rosiglitazone (9.3% in rosiglitazone group versus 5.1% in metformin group and 3.5% in glyburia group) [6]. In a prospective cohort study, 4 year-follow-up data from elderly American (mean age > 70 years) showed that treatment with TZDs including troglitazone, pioglitazone, and rosiglitazone was associated with additional whole-body bone loss in older diabetic women [7]. Moreover, the side effects of TZDs on bone metabolism were demonstrated in animal experiments as well. Trabecular bone volume and bone mineral density (BMD) were found to be decreased in rosiglitazone-treated mice [8–11]. 

In humans, several randomized controlled trials were performed to observe the changes of biochemical markers of bone metabolism after treatment with rosiglitazone. The results indicated that the levels of bone formation markers were significantly decreased in healthy or diabetic postmenopausal women, whereas the levels of bone resorption markers remained unchanged [12, 13]. On the other hand, several *in vitro* studies demonstrated that TZDs increased the allocation of mesenchymal stem cells toward adipocytes and decreased differentiation toward osteoblasts, and therefore inhibited osteoblastogenesis [14–16]. Although the adverse effects of TZDs on bone metabolism are well known and possibly attributable to the inhibition of bone formation, the exact mechanism of the effects is little known and needs to be elucidated. Moreover, the majority of available clinical data about the effects of TZDs on bone metabolism come from rosiglitazone, and the data from pioglitazone are very limited. In this regards, we designed the present study to investigate the effects of pioglitazone on biochemical markers of bone turnover in patients with T2DM. 

## 2. Materials and Methods

Seventy patients with T2DM, including 33 males and 37 females, were screened and enrolled in this study. Ten cases out of 37 females were premenopausal, while 27 cases were postmenopausal with mean menopausal duration of 5.9 ± 2.6 years. Inclusion criteria were 25 to 70 years of age, 19 to 35 kg/m^2^ of body mass index (BMI), previous therapy regimen including lifestyle modification with or without oral antihyperglycemic agents (monotherapy or combination therapy for at least 2 months), and fasting plasma glucose >7.0 mmol/L and ≤13 mmol/L. The patients were also required to be free of diabetic symptom, diabetic ketoacidosis, nonketotic hyperosmolar coma, renal dysfunction (serum creatinine levels >136 mmol/L), hepatic dysfunction (serum alanine aminotransferase or aspartate aminotransferase >2 times over the upper limit of normal range), and severe heart disease. Additionally, patients receiving previous therapy of any TZDs and pregnant or breast-feeding women or taking oral contraceptive pills were excluded from this study. The patients treated by vitamin D and/or bisphosphonate were also excluded. The study protocol was approved by the Ethical Committee of Peking University Health Science Center. All subjects gave informed consent. 

Pioglitazone (East China Pharmaceutical Company, Hangzhou, China) 15–45 mg once daily was added on the previous therapy regimen for 12 weeks in all eligible patients, and the previous antihyperglycemic agent, if any, should remain unchanged. Pioglitazone dosage was adjusted according to the fasting blood glucose levels tested once every 4 weeks. All subjects underwent complete clinical examinations and laboratory tests at baseline and at the end of the study. Laboratory tests included the measurement of fasting plasma glucose, glycosylated hemoglobin (HbA_1c_), lipid profiles, serum calcium, and phosphate, as well as serum levels of bone formation markers, procollagen type 1 N-terminal propeptide (P1NP), osteocalcin (OC), total alkaline phosphatase (BAP), and bone resorption marker, C-terminal telopeptide of type 1 collagen (CTX). 

Serum P1NP was analyzed by competitive RIA (Uniq-P1NP RIA, Orion Diagnostica, Espoo, Finland); Serum bone specific ALP (BAP) and Serum OC and Serum CTX were determined by ELISA (IDS Ltd, Boldon, UK). The intra- and interassay coefficients of variation were as follows: BAP intra-assay CV 2.6%–6.5% and interassay CV 3.7%–6.4%; P1NP intraassay CV 6.5%–10% and inter-assay CV 6.0%–9.8%; OC intraassay CV 1.3%–2.2% and interassay CV 2.5%–7.1%; CTX intraassay CV 1.7%–3.0% and interassay CV 2.5–10.9%. Plasma glucose levels were measured using the glucose-oxidase method, while HbA_1c_ values were assessed by high-performance liquid chromatography (HPLC). Serum lipid profiles, calcium, and phosphate were analyzed by automatic biochemical analyzer. 

Data were expressed as mean ± SD. Statistical analysis for the comparisons of mean values was performed using paired Student's *t*-test. Because the values of serum P1NP, OC, and CTX did not follow the Gaussian distribution, the values were presented as median (interquartile range) and comparisons were carried out using Wilcoxon signed-rank test as appropriate. Logarithmic (log) transformation of P1NP, OC, and CTX values was carried out before performing correlation analysis. Statistical analysis was carried out using the Statistical Program for Social Sciences (Version 20.0; SPSS Inc., Chicago, IL, USA). The two-tailed value of *P* < 0.05 was considered statistically significant.

## 3. Results 

Seventy patients, including 33 males and 37 females with T2DM, were enrolled in and completed the study. The mean age was 53.6 ± 8.8 years. The mean duration of disease was 6.0 ± 4.6 years. At baseline, the age, BMI, and diabetic duration showed no statistically significant differences between male and female subgroups. 

### 3.1. Changes in Metabolic Profiles after Pioglitazone Treatment

Compared to those before pioglitazone treatment, the levels of fasting plasma glucose, HbA_1c_, triglycerides, and diastolic blood pressure were significantly decreased after 12 weeks of pioglitazone treatment in the overall T2DM patients (*P* < 0.05). However, BMI, total cholesterol, and low-density lipoprotein cholesterol (LDL-C) levels were significantly increased after 12 weeks of pioglitazone treatment (*P* < 0.05). Furthermore, there were no significant differences in all metabolic parameters including BMI, blood glucose control, blood pressure, and lipid profiles between male and female subgroups at the end of 12-week treatment with pioglitazone (Table 1). 

### 3.2. Effects of Pioglitazone on Biochemical Markers of Bone Turnover

Compared to baseline, both serum BAP and P1NP levels declined markedly after 12 weeks of pioglitazone treatment in the overall T2DM patients (*P* < 0.01). It was noteworthy that serum BAP and P1NP levels were significantly decreased after pioglitazone treatment in the female subgroup rather than in the male subgroup. Although serum levels of BAP and P1NP had a trend to decline after pioglitazone treatment, there were no significant differences in the male subgroup. In addition, no statistically significant changes were observed in serum OC, CTX, calcium, and phosphate levels after pioglitazone treatment (Table 1).

### 3.3. Subgroup Analyses of Bone Turnover Markers after Pioglitazone Treatment in Patients with Type 2 Diabetes

In order to investigate whether menopause in female or the age factor in male affect the bone turnover markers after pioglitazone therapy, subgroup analyses were conducted. 37 female patients were divided into two subgroups, premenopause subgroup (*n* = 10) and postmenopause subgroup (*n* = 27). Similarly, the 33 male patients were also divided into two subgroups, age <50 years (*n* = 14) and age ≥50 years. Subgroup analysis showed that in postmenopause subgroup the levels of serum BAP and P1NP were significantly decreased after pioglitazone treatment, compared with those before pioglitazone treatment (Table 2). However, the bone turnover markers remained unchanged in two male subgroups after pioglitazone treatment (Table 3).

### 3.4. Correlation Analysis of Relationships between Baseline Values of Demographic and Biochemical Parameters versus Baseline Bone Turnover Markers or Changes in Bone Turnover Markers after Pioglitazone Treatment

Correlation analysis revealed that only changes in HbA_1c_ are negatively correlated with the changes in CTX (*r* = −0.360, *P* = 0.002) rather than changes in BAP or P1NP after pioglitazone therapy, while age, BMI, baseline glucose, HbA_1c_, and lipid profiles are not correlated with changes in BAP, P1NP, or OC.

## 4. Discussion 

The present study demonstrated that pioglitazone treatment could significantly decrease the serum concentrations of P1NP and BAP, two of biochemical markers of bone formation, in the overall patients with T2DM. In addition, the detrimental effects found in the overall patients should be attributable to decreased serum P1NP and BAP levels in the postmenopausal female patients rather than in premenopausal female patients or male patients. The results of correlation analysis showed that the changes in bone turnover markers were not associated with the changes in HbA_1c_ by pioglitazone treatment, suggesting independent effects of pioglitazone on bone turnover.

Pioglitazone plays a critical pharmacological role as regulators of glucose homeostasis, lipid metabolism, and cell proliferation by activating the nuclear receptor PPAR-*γ* [17]. In 2007, Takeda pharmaceutical company declared that long-term treatment with pioglitazone increased the incidence of fracture in women with T2DM. This declaration hinted that pioglitazone may have clinically important adverse effects on bone. The data from Health ABC Study also suggested that treatment with TZDs, including pioglitazone, rosiglitazone and troglitazone, was associated with greater bone loss at the whole body, lumbar spine, and trochanter in women, but not men, with diabetes [7]. Owing to limited information available in human diabetes studies, the mechanism regarding the effects of TZDs on human bone is little known and needs to be clarified. In a small clinical study of troglitazone, urine type 1 collagen *N-*telopeptide and serum bone-specific alkaline phosphatase were reduced after the first month of treatment but returned to baseline levels by 12 months of treatment [18]. By now, a few studies have reported the effects of pioglitazone on bone metabolic markers in patients with diabetes. It is described that pioglitazone decreased significantly the alkaline phosphatase and induced on average a 45% increase in urinary calcium excretion [19]. Decreased OC after 6-month treatment with pioglitazone also was reported although the change at 3 months was not significant [20]. In our study, we found that serum levels of BAP and P1NP, but not OC, were significantly reduced after 12-week treatment with pioglitazone in the overall T2DM patients, suggesting decreased osteoblast activity. The present data are in agreement with a pioglitazone study in polycystic ovary syndrome (PCOS) [21]. Similar results were also seen in some troglitazone studies [16, 22, 23]. The effects of pioglitazone on different bone formation markers may be related to their expression at different stages of osteoblastic differentiation. Both BAP and P1NP are markers of early bone formation. The former is representative early marker of osteoblastic differentiation and bone formation during the matrix maturation phase, and the latter is product of extracellular processing of procollagen before fiber assembly and appears during osteoblast proliferation. OC, however, is expressed in mature osteoblasts and involved in the arrangement of the mineral phase in bone. In addition, our findings are supported by several basic studies. *In vitro* studies show that activation of PPAR-*γ* signaling by pioglitazone or other TZDs could promote differentiation of pluripotent mesenchymal stem cells into adipocytes at the expense of osteoblasts and suppress osteoblast differentiation and OC expression in osteoblastic cell lines [14, 24]. Taken together, the present study and other reported studies suggested that pioglitazone or other TZDs may affect bone formation at an earlier stage and eventually reduce bone mineral density. Inhibition of bone formation could be responsible for TZDs-induced human bone loss. 

CTX is well known as an important biochemical marker of bone resorption. In the present study, serum CTX level was not significantly changed after 12-week treatment with pioglitazone, which was supported by a clinical study in obese premenopausal patients with PCOS [21]. In addition, an *in vitro* study showed that pioglitazone did not stimulate bone resorption in cultured mouse calvarial bones [25]. These data suggested that pioglitazone may not affect bone resorption. Likewise, two randomized controlled trials demonstrated that the markers of bone resorption *β*-CTX and deoxypyridinoline were not altered either in healthy or in diabetic postmenopausal women after rosiglitazone administration [16, 22]. Although controversy exists with regard to the effects of TZDs on bone resorption in several *in vitro* studies, the data from human studies seemed to be consistent and suggested that rosiglitazone or pioglitazone had no effects on bone resorption. 

Interestingly, the present study showed that pioglitazone led to significant reduction of serum BAP and P1NP levels in female subgroup, but not in male subgroup of the T2DM patients, suggesting that the effects of TZDs on bone formation seemed to be sex-specific. This finding is supported by almost all of TZD clinical studies, including the ADOPT [6] and Health ABC Study [7]. Up to now, most of the human data showing harmful impact of TZDs on bone were consistently obtained from women, especially from older or postmenopausal women. Whether it is also the case in men remains unknown. In fact, there are only a few clinical studies available and the results were conflicted. However, recent evidences from several studies focused on male TZDs' user demonstrated that rosiglitazone was associated with significant decrease in BMD of both spine and hip, and with an increased prevalence of fractures in males with T2DM [26, 27]. Considering that bone turnover rate is greater in older women than in older men and that TZDs had been reported to cause a decrease in estrogen levels by inhibiting the aromatase pathway [28], older women seem more likely to be at higher risk of bone metabolic abnormalities than older men in response to treatment with TZDs. Nevertheless, due to the lack of large-scale trials focused on bone metabolic markers, whether the effects of TZDs on bone turnover are sex-specific or not remains to be clarified. 

In agreement with previous reports, the present study confirmed that pioglitazone as an add-on treatment to failing monotherapy or combine therapy is efficient in T2DM patients with poorly controlled glucose levels [29, 30]. Pioglitazone was also shown to affect lipid metabolism in our study where the levels of triglycerides were markedly decreased and LDL-C levels were slightly but significantly increased after pioglitazone treatment. In fact, the beneficial effect of pioglitazone on triglycerides has been well established, whereas the effects of pioglitazone on total cholesterol and LDL-C are still a little inconsistent in the literatures [31]. The reason why pioglitazone elevates LDL-C levels is unclear but is likely to represent an increase in LDL particle size [32]. 

Limitations of the present study are its short duration of trial and lack of a control group. Additionally, there was no BMD measurement for comparison due to the short term of followup. 

## 5. Conclusions

The present study suggests that pioglitazone reduces bone formation at earlier stages but may have no impact on bone resorption in the patients with T2DM. This effect was not associated with decrease in HbA_1c_ by pioglitazone treatment. The reduction in bone formation is speculated to be the main reason for bone metabolic abnormality in the diabetic patients receiving pioglitazone therapy. Moreover, our study also showed that the detrimental effect of pioglitazone on bone formation was more obvious in postmenopausal women than in men or in premenopausal women with T2DM. The potential side effects of pioglitazone on bone metabolism should be given more attention in clinical practice.

## Figures and Tables

**Table 1:** Changes of metabolic profiles and bone turnover markers after pioglitazone treatment.

	Overall (*n* = 70)		Male (*n* = 33)		Female (*n* = 37)	
	Before therapy	After therapy	Before therapy	After therapy	Before therapy	After therapy
Age (yr)	53.6 ± 8.8		52.3 ± 8.7		54.7 ± 8.8	
Duration (yr)	6.0 ± 4.6		6.3 ± 5.4		5.9 ± 3.8	
BMI (kg/m^2^)	26.8 ± 2.9	27.0 ± 2.9	26.7 ± 2.9	26.9 ± 2.8	26.8 ± 2.9	27.1 ± 3.0
FBG (mmol/L)	9.1 ± 1.6	7.9 ± 1.5^#^	9.2 ± 1.6	8.1 ± 1.8^#^	9.1 ± 1.7	7.8 ± 1.2^#^
HbA_1c_ (%)	8.2 ± 1.4	7.1 ± 1.0^#^	8.0 ± 1.1	7.0 ± 0.9^#^	8.3 ± 1.7	7.2 ± 1.1^#^
BMI (kg/m^2^)	26.8 ± 2.9	27.0 ± 2.9*	26.7 ± 2.9	26.9 ± 2.8	26.8 ± 2.9	27.1 ± 3.0
SBP (mmHg)	122.2 ± 12.0	121.9 ± 12.4	120.0 ± 13.3	120.0 ± 12.7	123.8 ± 11.5	123.2 ± 12.1
DBP (mmHg)	77.1 ± 7.5	74.5 ± 6.5*	77.0 ± 8.4	74.0 ± 6.3	77.6 ± 6.7	75.0 ± 6.8
T-CHO (mmol/L)	4.9 ± 0.8	5.3 ± 0.9^#^	4.7 ± 0.8	5.2 ± 1.0^#^	5.1 ± 0.8	5.4 ± 0.9*
TG (mmol/L)	2.4 ± 1.6	1.9 ± 1.1^#^	2.3 ± 1.3	2.1 ± 1.3	2.5 ± 1.8	1.7 ± 0.9^#^
HDL-C (mmol/L)	1.1 ± 0.3	1.1 ± 0.3	1.0 ± 0.2	1.0 ± 0.2	1.2 ± 0.3	1.2 ± 0.3
LDL-C (mmol/L)	3.1 ± 0.8	3.2 ± 0.9*	3.0 ± 0.7	3.2 ± 0.9*	3.2 ± 0.8	3.2 ± 0.9
Ca^2+^ (mmol/L)	2.4 ± 0.1	2.3 ± 0.1	2.4 ± 0.9	2.3 ± 0.8	2.4 ± 0.1	2.3 ± 0.1
P^3−^ (mmol/L)	1.2 ± 0.2	1.2 ± 0.2	1.1 ± 0.1	1.1 ± 0.1	1.3 ± 0.2	1.3 ± 0.1
BAP (*μ*gU/L)	13.2 ± 4.7	12.3 ± 5.0^#^	13.6 ± 6.0	13.2 ± 5.5	12.9 ± 3.8	11.5 ± 3.5^#^
P1NP (*μ*g/L)	40.1 ± 16.8	36.6 ± 15.3^#^	34.6 ± 10.2	32.1 ± 10.3	45.0 ± 20.0	40.6 ± 17.9^#^
OC (ng/mL)	10.5 ± 5.1	10.2 ± 4.7	9.5 ± 4.2	9.0 ± 3.3	11.5 ± 5.7	11.3 ± 5.5
CTX (ng/mL)	0.35 ± 0.20	0.35 ± 0.19	0.33 ± 0.13	0.31 ± 0.14	0.37 ± 0.24	0.39 ± 0.22

Data are mean ± SD. *P < 0.05; ^#^ *P* < 0.01, compared to before therapy. FBG: fasting blood glucose; BMI: body mass index; SBP: systolic blood pressure; DBP: diastolic blood pressure; T-CHO: total cholesterol; TG: triglycerides; HDL-C: high-density lipoprotein cholesterol; LDL-C: low-density lipoprotein cholesterol; BAP: bone-specific alkaline phosphatase; P1NP: procollagen type 1 N-terminal propeptide; OC: osteocalcin; CTX: C-terminal telopeptide of type 1 collagen.

**Table 2:** Subgroup analyses of bone turnover after pioglitazone treatment in female patients with type 2 diabetes.

	Premenopause (*n* = 10)		Postmenopause (*n* = 27)	
	Before therapy	After therapy	Before therapy	After therapy
Ca^2+^ (mmol/L)	2.3 ± 0.08	2.3 ± 0.1	2.3 ± 0.1	2.3 ± 0.07
P^3−^ (mmol/L)	1.1 ± 0.2	1.2 ± 0.2	1.3 ± 0.2	1.3 ± 0.1
BAP (*μ*gU/L)	13.7 ± 5.3	13.0 ± 4.6	12.6 ± 4.8	10.9 ± 3.6*
P1NP (*μ*g/L)	28.1 ± 8.5	25.0 ± 8.8	51.2 ± 19.4	46.4 ± 16.9*
OC (ng/mL)	6.1 ± 1.2	6.3 ± 2.1	13.2 ± 5.5	13.1 ± 5.1
CTX (ng/mL)	0.15 ± 0.06	0.18 ± 0.07	0.46 ± 0.23	0.47 ± 0.20

Data are mean ± SD. *P < 0.05, compared to before therapy. BAP: bone-specific alkaline phosphatase; P1NP: procollagen type 1 N-terminal propeptide; OC: osteocalcin; CTX: C-terminal telopeptide of type 1 collagen.

**Table 3:** Subgroup analyses of bone turnover after pioglitazone treatment in male patients with type 2 diabetes.

	<50 yrs (*n* = 14)		≥50 yrs (*n* = 19)	
	Before therapy	After therapy	Before therapy	After therapy
Ca^2+^ (mmol/L)	2.3 ± 0.1	2.3 ± 0.08	2.3 ± 0.07	2.3 ± 0.07
P^3−^ (mmol/L)	1.1 ± 0.1	1.2 ± 0.1	1.1 ± 0.1	1.1 ± 0.1
BAP (*μ*gU/L)	13.2 ± 5.7	13.0 ± 6.2	14.1 ± 5.9	13.5 ± 5.5
P1NP (*μ*g/L)	38.1 ± 9.5	35.8 ± 9.9	31.7 ± 10.0	29.1 ± 9.8
OC (ng/mL)	10.8 ± 4.8	9.9 ± 3.5	8.4 ± 3.2	8.2 ± 3.1
CTX (ng/mL)	0.37 ± 0.11	0.35 ± 0.14	0.29 ± 0.14	0.27 ± 0.14

Data are mean ± SD. BAP: bone-specific alkaline phosphatase; P1NP: procollagen type 1 N-terminal propeptide; OC: osteocalcin; CTX: C-terminal telopeptide of type 1 collagen.
